# Current perspectives, beliefs and practices concerning delirium in critically ill patients: a multicenter survey among Dutch healthcare professionals

**DOI:** 10.1186/cc12335

**Published:** 2013-03-19

**Authors:** Z Trogrlic, M Van der Jagt, P Van der Voort, J Bakker, E Ista

**Affiliations:** 1 Erasmus MC University Medical Center, Rotterdam, the Netherlands

## Introduction

The beliefs, knowledge and practices regarding ICU delirium among ICU professionals may vary. This may interfere with the implementation of the Dutch ICU Delirium guideline. We aimed to get insight into potential barriers and facilitators for delirium guideline implementation that may help to find an effective implementation strategy.

## Methods

An online survey was sent to healthcare professionals from the six participating ICUs. Respondents included ICU physicians, nurses and delirium experts (psychiatrists, neurologists, geriatricians, nurse experts). The survey consisted of statements on beliefs, knowledge and practices towards ICU delirium. Agreement with statements by more than 75% of respondents were regarded as facilitating items and agreement lower than 50% as barriers for implementing protocolled care.

## Results

Of the 565 surveys distributed, 360 were completed (63.7%). The majority of respondents were ICU nurses (79%). Delirium was considered a major problem (83%) that requires adequate treatment (99%) and is underdiagnosed (81%). Respondents considered that routine screening of delirium can improve prognosis (95%). However, only a minority (20%) answered that delirium is preventable. Only 39% of the respondents had received any training about delirium in the previous 3 years and 77% of them found training useful. The mean delirium knowledge score was 6.6 out of 10 (SD = 1.54). When all groups were mutually compared, nurses scored lower than delirium experts (ANOVA, *P = *0.013). The respondents (58%; *n = *210) from three ICUs indicated that CAM-ICU assessment was department policy. However, 50% (*n = *106) of these respondents felt unfamiliar with CAMICU and only 47% (*n = *99) of them indicated that a positive CAM-ICU was used for treatment decisions. Haloperidol was the first-choice pharmacological treatment. Only 21% of all respondents knew that a national ICU delirium guideline existed, but in-depth knowledge was generally low.

## Conclusion

Our survey showed that healthcare professionals considered delirium an important but underdiagnosed form of organ failure. In contrast, screening tools for delirium are scarcely used, knowledge can be improved and protocolled treatment based on positive screening is often lacking. These results suggest that the focus of implementation of ICU delirium management should not be on motivational aspects, but on knowledge improvements, training in screening tools and implementation of treatment and prevention protocols.

