# Correction: Current perspectives, beliefs and practices concerning delirium in critically ill patients: a multicenter survey among Dutch healthcare professionals

**DOI:** 10.1186/cc12704

**Published:** 2013-05-10

**Authors:** Z Trogrlic, M van der Jagt, P van der Voort, H Ponssen, J Schoonderbeek, F Schreiner, S Verbrugge, S Duran, J Bakker, E Ista

**Affiliations:** 1 Erasmus University Medical Center, Department of Intensive Care, Rotterdam, The Netherlands; 2 Onze Lieve Vrouwe Gasthuis (OLVG), Department of Intensive Care, Amsterdam, The Netherlands; 3 Department of Intensive Care, Albert Schweitzer Hospital, Dordrecht, The Netherlands; 4 Department of Intensive Care, Ikazia Hospital, Rotterdam, The Netherlands; 5 Department of intensive care, IJsselland Hospital, Rotterdam, The Netherlands; 6 Department of Intensive Care, Sint Franciscus Gasthuis, Rotterdam, The Netherlands; 7 Department of Intensive Care, Maasstad Ziekenhuis, Rotterdam, The Netherlands

## Correction

After the publication of this abstract [1], we found that not all authors were listed. A full author list is provided here. We regret any inconvenience this error may have caused.
